# The role of bile acid metabolism-related genes in prognosis assessment of hepatocellular carcinoma and identification of NPC1 as a biomarker

**DOI:** 10.3389/fendo.2025.1588529

**Published:** 2025-05-29

**Authors:** Maosen Xu, Yu Zhang, Yan Tie, Yiqiao Luo, Yang Wang, Ziqi Zhang

**Affiliations:** ^1^ Laboratory of Aging Research and Cancer Drug Target, State Key Laboratory of Biotherapy, National Clinical Research Center for Geriatrics, West China Hospital, Sichuan University, Chengdu, Sichuan, China; ^2^ Department of Biotherapy, Cancer Center, West China Hospital, Sichuan University, Chengdu, China; ^3^ Cancer Center, West China Hospital, Sichuan University, Chengdu, Sichuan, China; ^4^ Department of Medical Oncology, National Cancer Center/National Clinical Research Center for Cancer/Cancer Hospital, Chinese Academy of Medical Sciences and Peking Union Medical College, Beijing, China

**Keywords:** hepatocellular carcinoma, bile acids metabolism, NPC1, tumor microenvironment, immune

## Abstract

Hepatocellular carcinoma (HCC) is one of the most prevalent and deadly cancers worldwide, with a high recurrence rate and poor prognosis. Understanding the molecular mechanisms driving HCC progression is crucial for improving prognostic accuracy and developing targeted therapies. Bile acids (BAs), as critical regulators of liver metabolism and inflammation, have recently been implicated in tumorigenesis and cancer progression. In particular, bile acids metabolism (BAM)-related genes play a pivotal role in the regulation of cellular proliferation, apoptosis, and immune responses in HCC. In this study, we explored the prognostic significance of BAM-related genes in HCC. Using a comprehensive bioinformatics approach, we analyzed transcriptomic data from public databases, identifying 111 differentially expressed BAM-related genes associated with patient survival. We then constructed a prognostic model based on these key genes, utilizing multivariate Cox regression analysis to determine their independent predictive value for overall survival in HCC patients. We identified four key BAM-related genes including AKR1D1, CYP7A1, FABP6, and NPC1 as significant prognostic markers. Among these genes, only NPC1 was the highly expressed gene and demonstrated statistically difference between HCC and normal liver tissues. The downregulation of NPC1 inhibited HepG2 cell proliferation, migration, and invasion. In conclusion, BAM-related genes offer a promising avenue for improving prognosis assessment in HCC patients. Our findings highlight the potential of NPC1 as a valuable tool for risk stratification and personalized treatment strategies in HCC patients.

## 1 Introduction

Liver cancer is the sixth most commonly diagnosed malignancy and the third leading cause of cancer-related death globally (1). Hepatocellular carcinoma (HCC) is the major form and accounts for more than 80% of liver cancers (2). Although tremendous progress has been achieved in the field of cancer therapy, the prognosis and five-year survival rate of patients with HCC remain unsatisfactory. Therefore, it is urgent to explore novel molecular markers associated with the progression of HCC so as to complement existing treatment regimens.

Bile acids (BAs), as critical regulators of liver metabolism and inflammation, have recently been reported to participate in tumorigenesis and cancer progression (3). Specifically, BAs are the primary metabolic byproducts of cholesterol in the liver and play crucial roles in regulating glucose, lipid, and energy metabolism. Beyond their physiological activities, BAs also function as signaling molecules and key regulators, modulating critical cellular processes including proliferation, apoptotic pathways, and immune responses in HCC (4). Simultaneously, previous clinical cohort studies have demonstrated that aberrant alterations in BAs profiles are closely related to HCC development (5, 6).

Mechanically, the diverse types of BAs have been demonstrated to influence the composition and functionality of the gut microbiota, and the bidirectional interaction between BAs and gut microbiota is associated with HCC progression (7). A previous study have revealed that chenodeoxycholic acid, a primary BA, contributes to HCC progression by mediating inflammasome activation via targeting mitochondrial reactive oxygen species overaccumulation (8). Moreover, the study conducted by Qu et al. found that impaired BAM is significantly associated with poor prognosis and advanced disease progression in patients with HCC. Furthermore, they proposed a novel molecular classification system, suggesting that HCC can be stratified into proliferative and non-proliferative subtypes based solely on the expression profiles of BAM-related genes (9). All the above findings provided new directions for HCC treatment by targeting dysregulated BAM-related genes in HCC.

The tumor microenvironment (TME) comprises diverse cellular components such as fibroblasts, endothelial cells, immune cells, and adipocytes, along with non-cellular elements such as stromal proteins, extracellular matrix, and numerous growth factors. These components collectively create a unique metabolic landscape that supports tumor progression through promoting cancer cell growth and inhibiting antitumor immunity (10–12). The functional differentiation and phenotypic plasticity of immune cells within the TME are usually affected by BAM. Aberrant BAM was demonstrated to inhibit the recruitment of natural killer T cells into the TME and promote M2-like tumor-associated macrophage polarization, thereby facilitating tumor immune evasion and HCC progression (13). The accumulation of both primary conjugated and secondary BAs represents a distinct metabolic hallmark in human HCC. Inhibition of conjugated BAs synthesis in hepatocytes significantly potentiated tumor-specific T cell immunity, suppressed hepatocarcinogenesis, and enhanced tumor susceptibility to anti-PD-1 therapy (14).

In the present study, we developed a polygenic prognostic signature utilizing TCGA cohort for HCC, followed by integrative bioinformatics analysis to assess the correlation between the prognostic model and tumor immune microenvironment characteristics along with clinical parameters. Subsequently, we constructed a prognostic model using LASSO regression and identified a four-gene signature comprising AKR1D1, CYP7A1, FABP6, and NPC1. Moreover, we also conducted *in vitro* functional studies to experimentally validate the role of NPC1 gene in HCC progression, with the aim to provide novel biomarkers for HCC detection and treatment.

## 2 Methods

### 2.1 Data acquisition

We retrieved RNA-sequencing data, normalized as fragments per kilobase of transcript per million mapped reads, along with the corresponding clinical and prognostic information from The Cancer Genome Atlas (TCGA) portal (http://cancergenome.nih.gov). The dataset was consist of 374 tumor samples and 50 adjacent normal tissue samples. Furthermore, we obtained the HCC dataset GSE14520 from the Gene Expression Omnibus (GEO) database for external validation, including 247 tumor samples and 241 adjacent normal tissue samples. To identify gene sets associated with bile acid and BAM, we utilized three gene sets of the Molecular Signatures Database (MsigDB, https://www.gsea-msigdb.org/gsea/msigdb), included the “HALLMARK_BILE_ACID_METABOLISM”, “REACTOME_BILE_ACID_AND_BILE_SALT_METABOLISM”, and “BILE_ACID_AND_BILE_SALT_METABOLISM”. A total of 139 genes are primarily involved in BAs and BAM.

### 2.2 Differential expression analysis and construction of consensus clustering

Differentially expressed genes (DEGs) analysis was conducted by the “limma” package of R language on transcriptomic data obtained from the TCGA database. The statistical significance threshold was set at p-value < 0.05 and |log_2_FC| > 0.5. Through this rigorous analytical approach, a total of 111 DEGs of BAM meeting these criteria were identified and selected for further investigation. In addition, the expression data of 111 BAM-related genes were extracted from TCGA cohort. The R package of ConsensusClusterPlus was used to perform subsequent consensus unsupervised sample clustering (15). To improve the reliability and stability of the Clustering results, an iterative clustering approach was implemented with 50 independent replicates.

### 2.3 GSEA and mutation analysis

Gene Set Enrichment Analysis (GSEA) was performed to quantify the enrichment of specific pathways or functional features across different clusters. The analysis was conducted using the R packages “limma”, “org.Hs.eg.db”, “clusterProfiler”, and “enrichplot”. Two gene set collections (c5.go.symbols.gmt and c2.cp.kegg.symbols.gmt) were utilized in the GSEA analysis. Statistical significance was determined using a threshold of p-value < 0.05 and false discovery rate (FDR) < 0.05. And the top 5 were shown.

To classify somatic mutation profiles in Mutation Annotation Format, Cluster group and risk score were conducted. Tumor mutational burden (TMB) was calculated from somatic mutation data for each patient. Comprehensive mutation analysis and generation of waterfall plots were conducted by the “maftools” R package. Moreover, Kaplan-Meier survival analysis and visualization of survival curves were performed with the “survival” and “survminer” R packages, respectively.

### 2.4 Immunity analysis of consensus clustering

For tumor immune analysis, the immune score, stromal score, and estimate score were computationally derived using the ESTIMATE algorithm as previously used (16). The computational analysis and visualization of ESTIMATE algorithm results derived from TCGA database were performed using the following R packages: “limma”, “estimate”, and “ggpubr”. The infiltration profiles of 22 immune cell subtypes across different sample groups were assessed using the CIBERSORT algorithm (17). To characterize immune cell infiltration patterns, we employed seven established algorithms: XCELL, TIMER, QUANTISEQ, MCPCOUNTER, EPIC, and CIBERSORT-ABS. The following essential R packages including “limma”, “scales”, “ggplot2”, “ggtext”, “reshape2”, “tidyverse” and “ggpubr” were used to conduct related analysis.

### 2.5 Construction of BAM-related genes prognosis model

To identify prognostic BAM-related genes, univariate Cox regression analysis was initially performed in conjunction with survival analysis. And then, the TCGA cohort served as the training dataset, while the GSE14520 cohort was utilized as the validation dataset. Based on the identified prognostic BAM-related genes, LASSO-Cox regression analysis was implemented to select optimal genes and construct a risk prediction model, thereby reducing the risk of overfitting. In the risk score calculation, Exp (Gene) represents the expression level of BAM-related genes, and Coef (Gene) denotes the corresponding regression coefficients. Survival analysis and visualization were conducted using the “survival” and “survminer” R packages to generate Kaplan-Meier survival curves.

### 2.6 Nomogram and calibration of prognosis model

The predictive nomogram was developed using R statistical software. Besides, the following packages were also used including “survival”, “regplot” and “rms”. Furthermore, the calibration curve was used to evaluate and quantify the concordance between predicted probabilities and observed outcomes across three clinically relevant time points (1-, 3-, and 5-year overall survival).

### 2.7 Survival analysis and clinical correlation analysis of prognosis model

The prognostic significance of four BAM-related genes was evaluated through survival analysis, with survival curves generated using R-based packages. The association between methylation status of four BAM-related genes and patient survival outcomes was analyzed using R statistical software. Survival curves were generated utilizing the “survival” and “survminer” package.

### 2.8 Cell culture and transfection

The HCC cell line HepG2 was purchased from ATCC(American Type Culture Collection) and was cultured in DMEM (Dulbecco’s Modified Eagle Medium) with 10% FBS (Fetal Bovine Serum) and 1% penicillin-streptomycin at 37°C in 5% CO_2_. Transfection of HepG2 cells was initiated at a confluence of approximately 30%. Lipofectamine 2000 (Lip2000) was employed as the transfection reagent following the manufacturer’s protocol. Briefly, OPTI-MEM (Optimized Minimum Essential Medium) was individually mixed with siRNA and Lip2000, followed by a 5-minute incubation at room temperature. The two mixtures were then combined and allowed to incubate for an additional 15 minutes to form transfection complexes. The resulting mixture was subsequently added to complete DMEM medium without antibiotics. After 6~8 hours, the medium was replaced. The knockdown efficiency of NPC1 was evaluated after 24 hours of transfection.

### 2.9 RNA extraction and quantitative real-time PCR

According to manufacturer’s instructions, total RNA was extracted using the RNA isolation kit (Foregene, Chengdu, China). The RNA was reverse-transcribed into cDNA by the PrimeScript RT reagent kit (TaKaRa, Japan). And then qPCR was performed to assess relative mRNA expression. Data were analyzed using the 2^-ΔΔCt^ method. The primer sequences were listed in **Supplementary Table S2**.

### 2.10 CCK8 and colony formation assay

After successful transfection of HepG2 cells, approximately 3×10^3^ cells from both the control and knockdown groups were seeded in 96-well plates for CCK8 assay. Cell viability was detected by incubating the cells for 0, 24 and 48 hours respectively. After incubation, 10 μl CCK-8 solution together with 90 μl fresh medium were added into each well and the culture plate underwent incubation for 1~4 hours at 37°C. The OD (Optical Density) at 450 nm was detected by a microplate reader.

After successful transfection of HepG2 cells, approximately 500 cells from both the control and knockdown groups were seeded into 6-well plates for colony formation assay. Then, the medium was replaced every 3 days. After the incubation for 14 days, the plates were washed with PBS (Phosphate Buffered Saline) for 3 times, fixed with 4% polyoxymethylene and stained with 0.5% crystal violet. Finally, the images were taken after the plates dried using an Olympus microscope.

### 2.11 Transwell assays and wound healing assay

To detect the effect of the NPC1 gene on the migration and invasion of HepG2 cells, transwell assays were conducted using the 8μm pore-size transwell plate. Briefly, approximately 4×10^4^ HepG2 cells undergone successful transfection were mixed with 200 µL serum-free medium on the upper chambers inserted into a 24-well plate. The bottom chamber was contained with 600 µL complete medium. For invasion assessment, the upper chamber was precoated with 20% Matrigel and cells were seeded into the chamber as previously. After incubation for 48 hours, the upper chamber was removed, washed with PBS, fixed using 4% paraformaldehyde and stained by 0.5% crystal violet. Finally, the migration or invasion cells were imaged by an Olympus microscope. After successful transfection, HepG2 cells were seeded in six-well plates and were cultured until reached 100%. Then, a pipette tip was used to form a linear wound in the confluent monolayer. Meanwhile, the cell debris was removed by PBS. Wound healing images were photographed at 0, 24, and 48 hours, respectively.

### 2.12 Statistical analysis

All statistical analyses were performed with R Studio (Version 4.4.1) and GraphPad Prism (Version 10.1.0). In the present study, p-value<0.05 was considered statistically significant difference.

## 3 Results

### 3.1 Construction and validation of molecular subtyping of BAM

**Figure 1:**
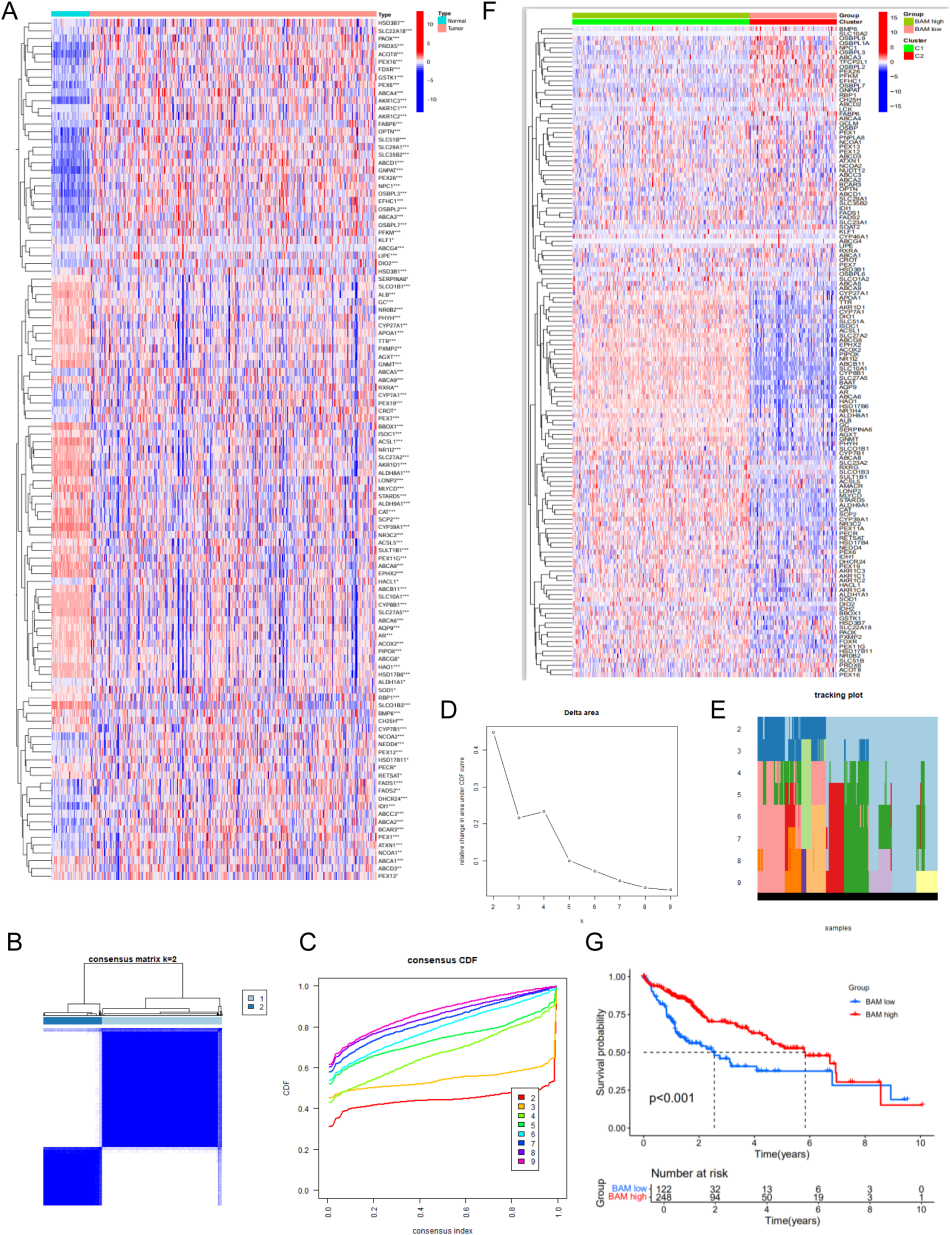
Identification of 111 BAM related-DEGs. **(A)** Identification of two subtypes based on Consensus clustering analysis. **(B)** Consensus clustering at k = 2. **(C)** The empirical cumulative distribution functions plotted for each k value. **(D)** Delta area diagram at different k **(E)** Longitudinal trajectory visualization for individual items across each k value. **(F)** The heatmap of 4,438 identified DEGs. **(G)** Survival analysis of HCC patients in two molecular subtypes. *p < 0.05, **p < 0.01, ***p < 0.001.

We first retrieved 138 BAM-related genes from the MSigDB database (**Supplementary Table S1**) and performed differential expression analysis using the TCGA cohort, identifying 111 differentially expressed genes(DEGs) (**Figure 1A**). Based on the aforementioned genes, we employed an unsupervised clustering methodology to construct molecular subtypes associated with BAM (**Figures 1B–E**). Additionally, our analysis identified 4,438 DEGs based on the molecular subtype (**Figure 1F**). Kaplan-Meier survival analysis demonstrated significantly reduced overall survival in patients with low BAM-related gene expression compared to the high counterparts (**Figure 1G**).

### 3.2 GSEA and mutation analysis

**Figure 2:**
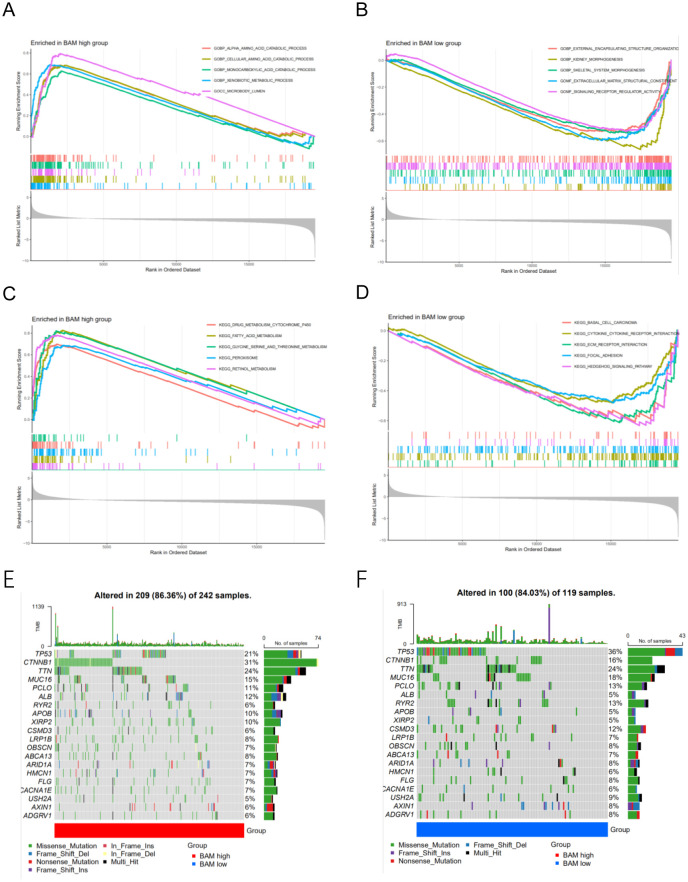
**(A)** GSEA of GO analysis in the high risk group. **(B)** GSEA of GO analysis in the low risk group. **(C)** GSEA of KEGG analysis in the high risk group **(D)** GSEA of KEGG analysis in the low risk group. **(E–F)** The mutation waterfall map of the high and low-risk group.

Through GSEA based GO, we observed that the BAM-high group is closely associated with GOBP_ALPHA_AMINO_ACID_CATABOLIC_PROCESS,GOBP_CELLULAR_AMINO_ACID_CATABOLIC_PROCESS, GOBP_MONOCARBOXYLIC_ACID_CATABOLIC_PROCESS, GOBP_XENOBIOTIC_METABOLIC_PROCESS, and GOCC_MICROBODY_LUMEN; BAM-low group is closely associated with GOBP_EXTERNAL_ENCAPSULATING_STRUCTURE_ORGANIZATIO,GOBP_KIDNEY_MORPHOGENESIS,GOBP_SKELETAL_SYSTEM_MORPHOGENESIS,GOMF_EXTRACELLULAR_MATRIX_STRUCTURAL_CONSTITUENT, and GOMF_SIGNALING_RECEPTOR_REGULATOR_ACTIVITY (**Figures 2A, B**). Through GSEA based KEGG, BAM-high group is closely associated with KEGG_DRUG_METABOLISM_CYTOCHROME_P450, KEGG_FATTY_ACID_METABOLISM, KEGG_GLYCINE_SERINE_AND_THREONINE_METABOLISM, KEGG_PEROXISOME and KEGG_RETINOL_METABOLISM; BAM-low group is closely associated with KEGG_BASAL_CELL_CARCINOMA,KEGG_CYTOKINE_CYTOKINE_RECEPTOR_INTERACTION, KEGG_ECM_RECEPTOR_INTERACTION, KEGG_FOCAL_ADHESION and KEGG_HEDGEHOG_SIGNALING_PATHWAY (**Figures 2C, D**).

Through mutation analysis, we observed differences in gene mutation frequencies between the high-risk and low-risk groups, particularly in TP53 and CTNNB1. The mutation frequency of TP53 was significantly lower in the high-risk group compared to the low-risk group, while CTNNB1 showed a significantly higher mutation frequency in the low-risk group compared to the high-risk group (**Figures 2E, F**). This finding provides a valuable therapeutic strategy.

### 3.3 Immune analysis based on molecular subtyping

**Figure 3:**
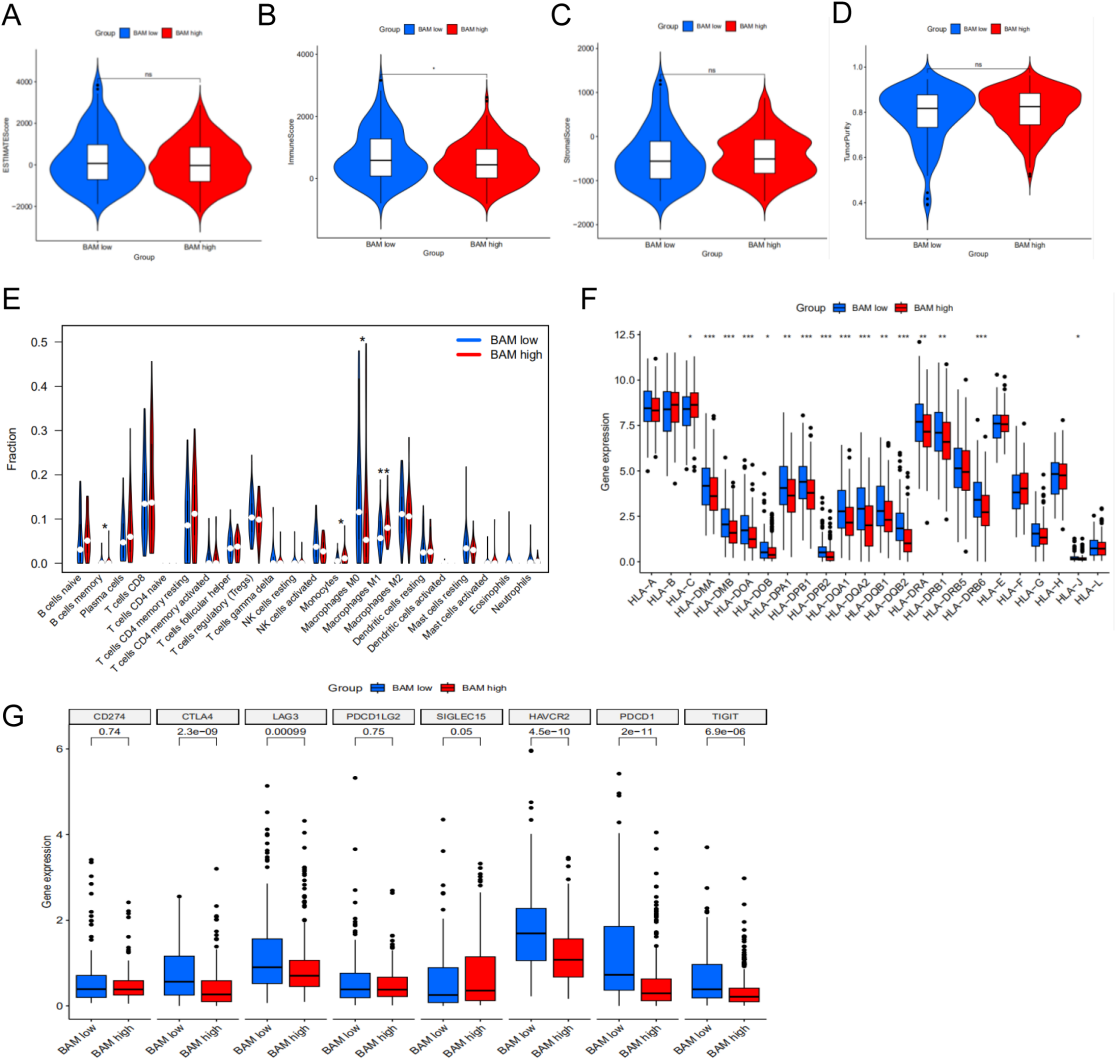
Immune cell infiltration in two molecular subtypes. **(A)** ESTIMATE Score in two subtypes. **(B)** Immune Score in two subtypes. **(C)** Stromal Score in two subtypes. **(D)** Tumor Purity Score in two subtypes. **(E)** The violin plot based on the CIBERSORT in two subtypes. **(F)** Differential expression patterns of HLA allelic variants between two molecular subtypes. **(G)** The difference between immune checkpoints in two subtypes. *p < 0.05**p < 0.01, ***p < 0.001.

Through comprehensive analysis of microenvironment scores, we observed a statistically significant difference in ImmuneScore, suggesting distinct immune microenvironment profiles between the two molecular subtypes (**Figures 3A–D**). Furthermore, we investigated the disparities in immune cell composition between the subtypes and identified memory B cells, monocytes, and M1 macrophages as the pivotal immune cell populations distinguishing the two molecular subtypes (**Figure 3E**). Lastly, we assessed the variations in HLA-related genes and immune checkpoint-related genes across the different molecular subtypes. Our analysis revealed several differentially expressed genes between the subtypes (**Figures 3F, G**).

### 3.4 Construction a prognosis signature based on BAM-related genes

**Figure 4:**
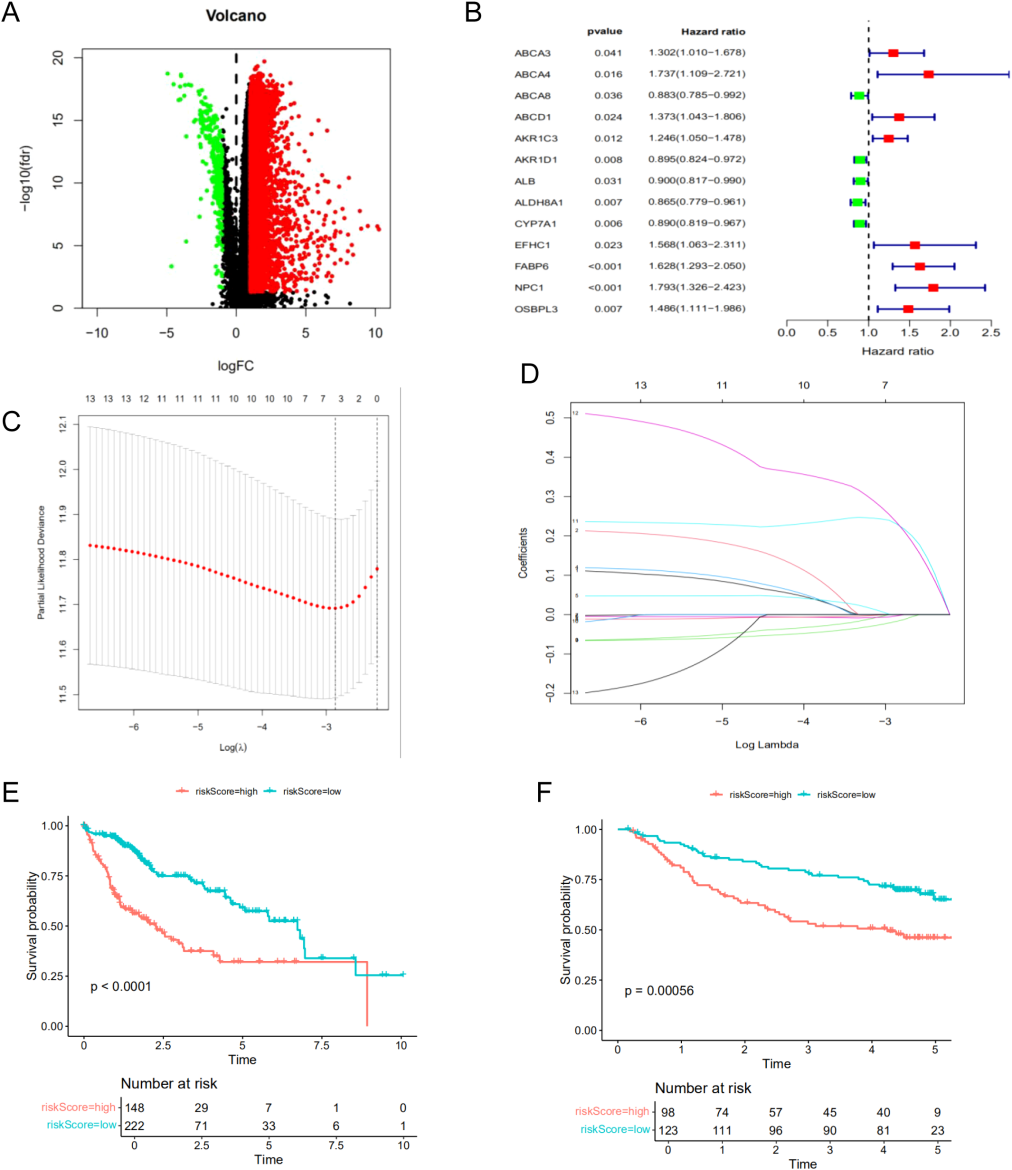
Construction of the prognostic model for HCC based on the screened BAM-related genes. **(A)** The volcano map of DEGs. **(B)** The univariate cox regression analysis of BAM-related genes. **(C, D)** The LASSO regression analysis and partial likelihood deviance on the prognostic genes signature. **(E)** Survival curve of the two risk groups based on the four-gene prognostic signature in the TCGA dataset. **(F)** Survival curve of the two risk subgroups defined by the four-gene signature in the GSE14520 cohort.

To construct a robust prognostic model for predicting patient outcomes, we utilized the TCGA cohort as the training set and the GSE14520 cohort from GEO as the validation set. Differential expression analysis was performed to identify common differentially expressed genes (DEGs) between the TCGA and GSE14520 cohorts, yielding a total of 5,069 DEGs (**Figure 4A**). Through univariate regression analysis, we identified 13 survival-related genes associated with BAM (**Figure 4B**). Subsequently, we constructed a prognostic model using LASSO regression, which identified a four-gene signature comprising AKR1D1, CYP7A1, FABP6, and NPC1 (**Figures 4C, D**). Survival analysis demonstrated a statistically significant difference between the high-risk and low-risk groups, with patients in the high-risk group exhibiting significantly shorter survival times compared to those in the low-risk group (**Figures 4E, F**).

**Figure 5:**
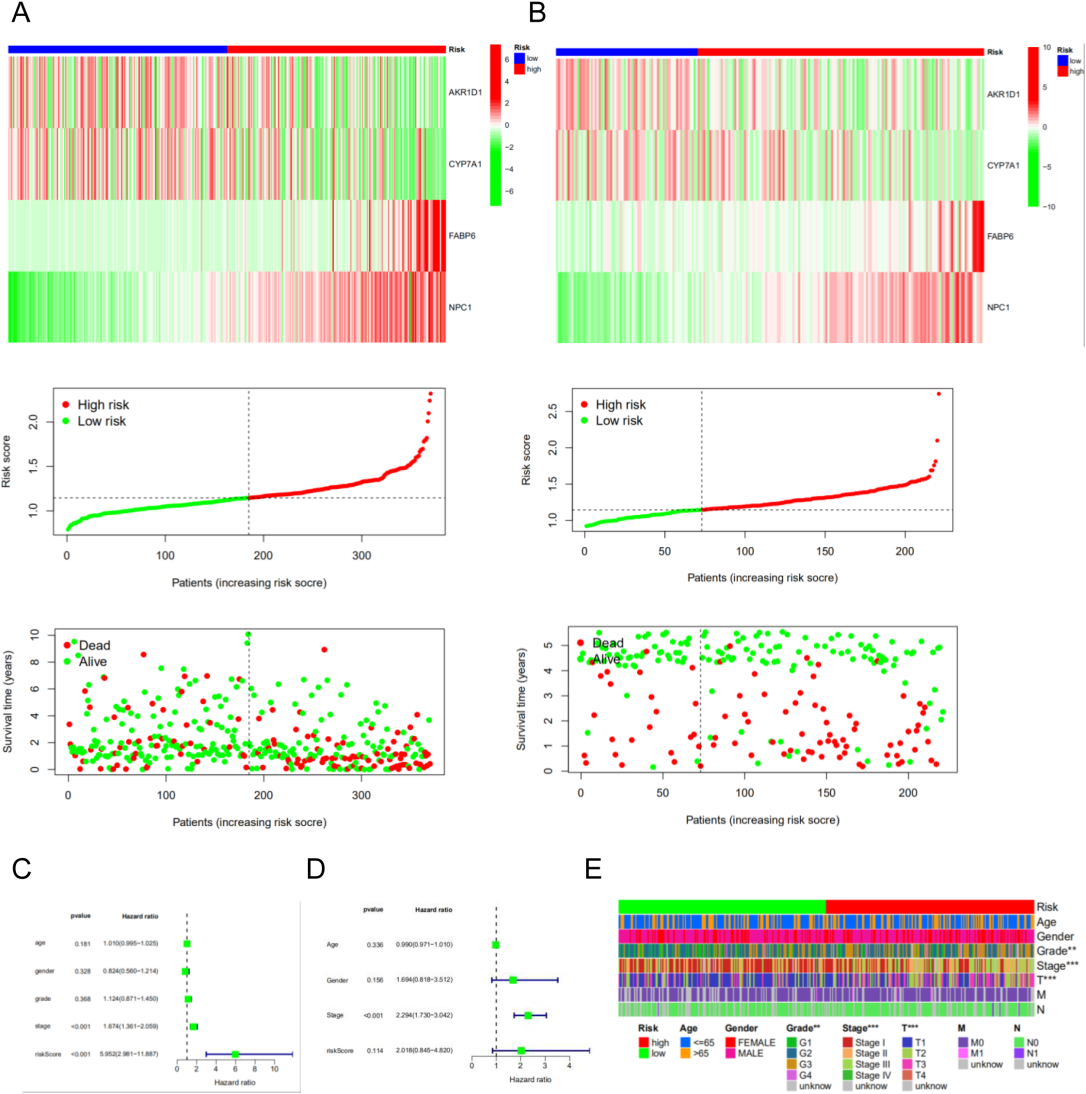
The risk model of four-gene signature. **(A)** The four-gene mRNA expression, risk score distribution, and the related survival data in the TCGA cohort. **(B)** The four-gene mRNA expression, risk score distribution, and the related survival data in the GSE14520 cohort. **(C, D)** The univariate and multivariate Cox regression analyses in the TCGA cohort and GSE14520 cohort. **(E)** The correlated heatmap of the risk score and clinical features.

For model evaluation, we employed heatmaps, survival time curves, and survival status plots, which collectively demonstrated the stability and robust prognostic performance of the four-gene model in both the TCGA cohort (**Figure 5A**) and the GSE14520 cohort (**Figure 5B**). Univariate and multivariate independent prognostic analyses demonstrated that the risk score can serve as an independent prognostic factor to predict the outcome in HCC patients within the TCGA cohort and the GSE14520 cohort. The univariate (**Figure 5C**) and multivariate Cox regression analyses (**Figure 5D**) in the TCGA cohort demonstrate that the risk score and stage are independent prognostic factors in HCC patients. Clinical correlation analysis revealed that riskscore is closely associated with grade, stage and T stage of TNM classification (**Figure 5E**).

### 3.5 Immune analysis of prognosis signature based BAM-related genes

**Figure 6:**
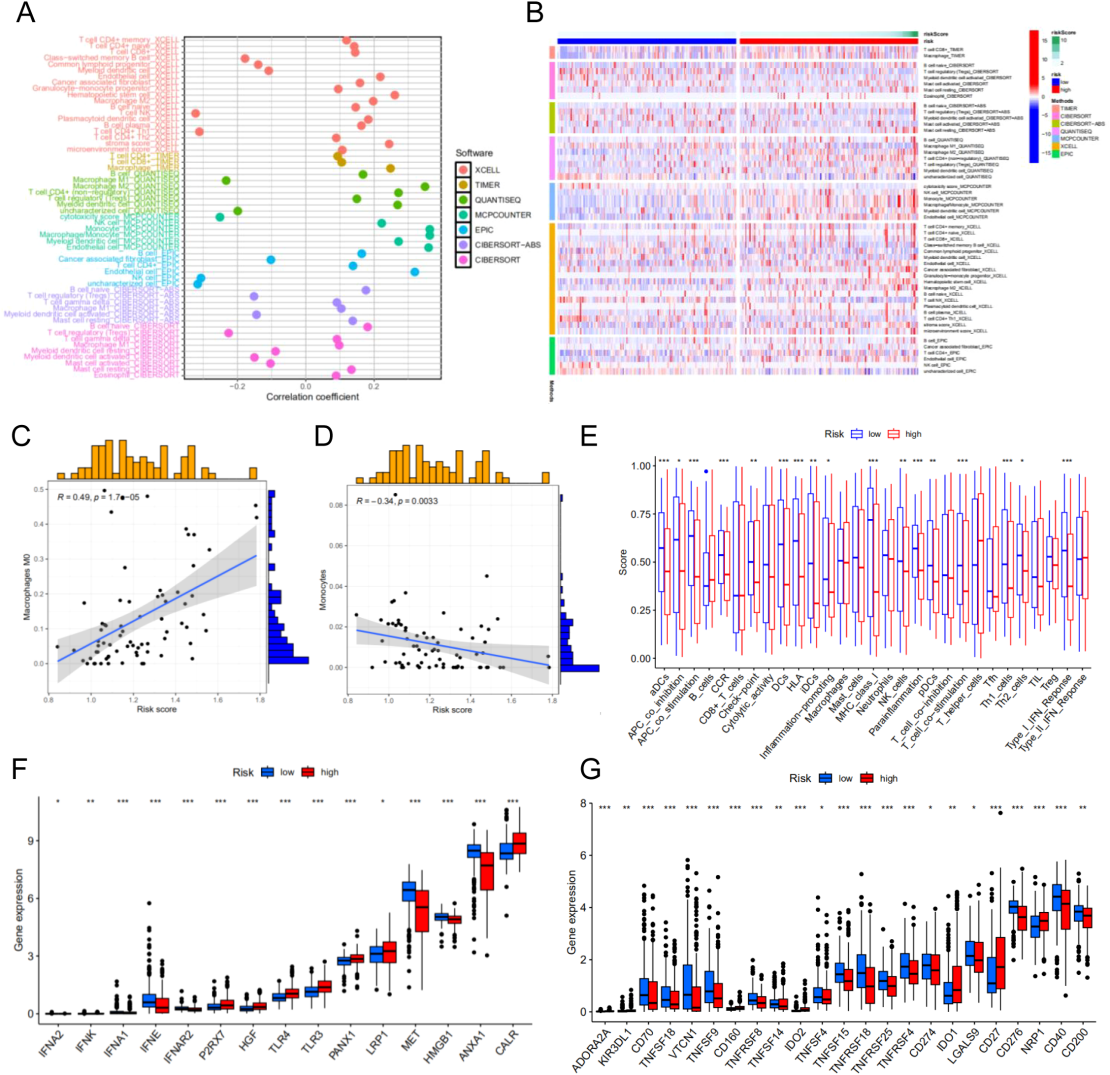
Immune cell infiltration analysis of different risk groups. **(A)** Correlation between risk scores and immune cells. **(B)** The distribution profiles of various immune cell subsets across samples with different risk scores. **(C)** The correlation between risk score M0 macrophages. **(D)** The correlation between risk score monocytes. **(E)** Immune function analysis-related risk score has associations with potential functional immune cells. **(F)** Correlation analysis between immune phenotypic clusters and ICD modulators. **(G)** Correlation analysis between immune phenotypic clusters and ICP modulators. *p < 0.05**p < 0.01, ***p < 0.001.

We perform the immune cell infiltration analysis. As illustrated in the bubble plot (**Figure 6A**), the quantitative assessment incorporating seven distinct computational methodologies - CIBERSORT, TIMER, QUANTISEQ, MCP-COUNTER, EPIC, xCell, and CIBERSORT-ABS - demonstrated statistically significant associations between risk stratification and tumor immune microenvironment composition. Our findings indicate a strong correlation between elevated risk scores and increased immune cell infiltration levels across multiple immune cell subtypes. Moreover, we employed heatmap to visualize the distribution profiles of various immune cell subsets across samples with different risk scores (**Figure 6B**). Based on the CIBERSORT analysis of the TCGA cohort, we observed that the risk score is significantly associated with Macrophages M0 and Monocytes (**Figures 6C, D**). Immune function analysis revealed differences in multiple immune cell functions between the high-risk and low-risk groups of the prognostic model (**Figure 6E**).

Immunogenic cell death (ICD) and immune checkpoint (ICP) molecules play pivotal roles in predicting the efficacy of immunotherapy in patients with malignancies (18, 19). Consequently, we assessed the differences in ICD- and ICP-related genes between the risk groups and identified statistically significant variations in multiple genes associated with these pathways. These findings suggest that ICP-based immunotherapy may yield differential responses in patients from distinct risk groups. Therefore, tailoring personalized immunotherapy strategies to account for the varying responses of different risk groups could potentially enhance therapeutic outcomes for patients with HCC (**Figures 6F, G**).

### 3.6 Construction and verification of the prognostic nomogram

**Figure 7:**
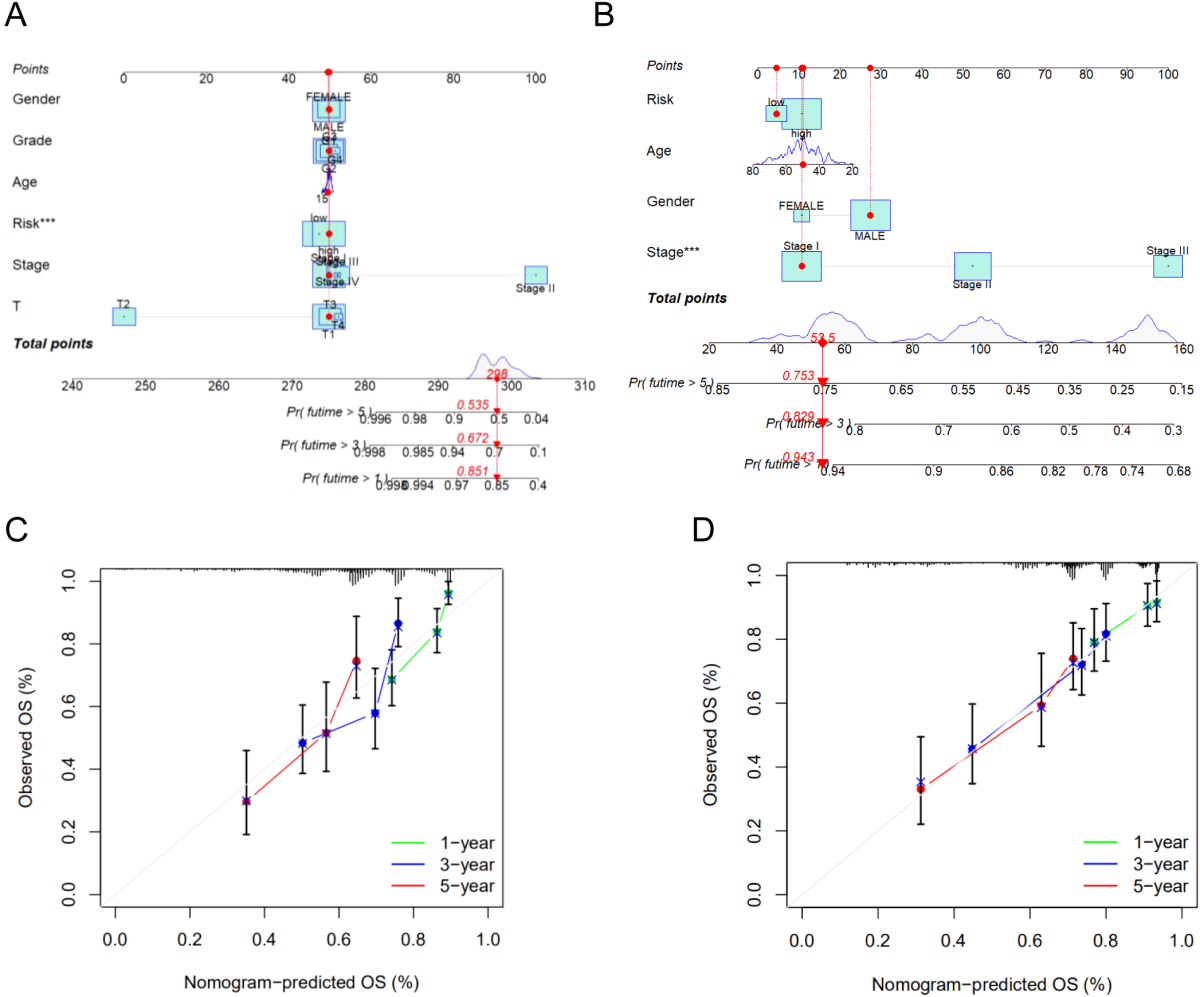
**(A)** The nomogram integrated the risk score and clinical features to predict the survival rate of the 1, 3, and 5 years in TCGA cohort. **(B)** The nomogram integrated the risk score and clinical features to predict the survival rate of the 1, 3, and 5 years in GSE14520 cohort. **(C)** The 1, 3, and 5 years OS calibration curves in TCGA cohort. **(D)** The 1, 3, and 5 years OS calibration curves in GSE14520 cohort. ***p < 0.001.

To further assess the predictive accuracy of the model for survival outcomes, we developed a nomogram incorporating age, gender, stage, and risk score using the TCGA cohort (**Figure 7A**) and the GSE14520 cohort (**Figure 7B**). Additionally, calibration plots were generated to evaluate the agreement between the nomogram-predicted and observed 1-, 3-, and 5-year survival rates (**Figures 7C, D**). The predicted survival curves demonstrated close alignment with the reference lines, indicating the reliability and accuracy of the constructed nomogram.

### 3.7 Validation of a four-gene prognostic model

**Figure 8:**
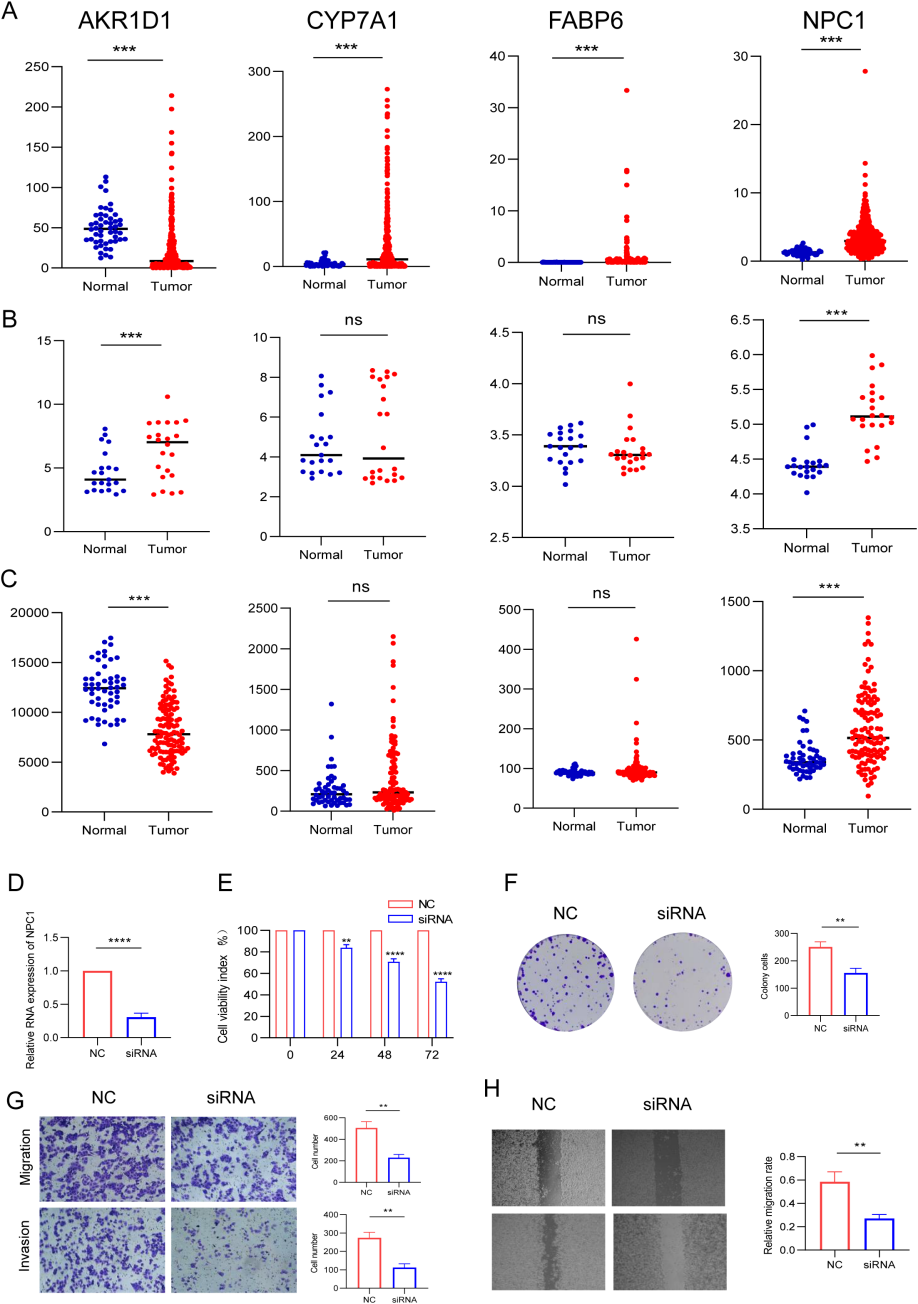
Differential expression analysis of the four BAM-related genes and *in vitro* validation. **(A)** TCGA. **(B)** GSE14520. **(C)** GSE76427. **(D)** The expression of NPC1 in HepG2 cell following siRNA transfection by assay. **(E, F)** Silencing NPC1 expression inhibited HepG2 cell proliferation and colony formation. **(G, H)** Silencing NPC1 expression inhibited the invasive and migratory capacity of HepG2 cell. **p < 0.01, ***p < 0.001, ****P < 0.0001..

We analyzed the expression of four BAM-related genes expression profiles across TCGA, GSE14520, and GSE76427 cohorts revealing distinct expression patterns between HCC tissues and normal liver tissues. The comparative analysis demonstrated that AKR1D1 expression was downregulated in HCC tissues compared to normal liver tissues. Conversely, three other BAM-related genes CYP7A1, FABP6 and NPC1 showed upregulation in HCC tissues relative to their expression levels in normal hepatic tissues. Among the above four genes, only NPC1 was the highly expressed gene and demonstrated statistical difference between HCC and normal liver tissues (**Figures 8A–C**). Therefore, we employed siRNA to downregulate NPC1 expression in HepG2 cells for further exploring its role in HCC pathogenesis. Through qPCR assay, we found that NPC1 was significantly downregulated following siRNA transfection (**Figure 8D**). Through CCK-8 assay, we found that NPC1 knockdown could inhibit cell proliferation and colony formation in HepG2 cells (**Figures 8E, F**). NPC1 knockdown also attenuated the migratory and invasive capacities of HepG2 cells by transwell assays (**Figure 8G**). Analogously, wound healing assay revealed that NPC1 knockdown impaired the migratory potential of HepG2 cell (**Figure 8H**). The above findings indicate that silencing NPC1 may suppress HCC progression, suggesting its potential utility as a novel diagnostic biomarker and therapeutic target for HCC.

## 4 Discussion

Hepatocellular carcinoma persists as one of the most therapeutically challenging malignancies, ranking as the sixth most prevalent cancer and the third leading cause of cancer-related mortality worldwide (20). Therapeutic approaches for HCC include non-drug and drug treatment. In early-stage HCC, the primary treatment strategies involve interventional procedures, including surgical resection, liver transplantation, and transarterial chemoembolization. For advanced HCC, systemic therapy predominantly relies on pharmacological interventions such as targeted agents and monoclonal antibody-based therapies (21, 22). Despite the availability of diverse therapeutic strategies for HCC, the clinical outcomes of patients remain suboptimal. To enhance patient prognosis, there is an urgent need to identify and validate novel prognostic biomarkers.

Bile acids include a variety of lipid compounds and are biosynthesized in the hepatic parenchyma and subsequently conjugated with sodium or potassium cations to form bile salts prior to their secretion into the gallbladder lumen. These primary BAs undergo microbial biotransformation into secondary BAs through the enzymatic activities of specific commensal microorganisms within the intestinal microbiota (23, 24). It is generally accepted that BAs exert pleiotropic effects on cellular physiology, modulating metabolic homeostasis, inflammatory responses, and proliferative signaling pathways through their interactions with specific nuclear and membrane-bound receptors. Emerging evidence increasingly demonstrates that, beyond their fundamental roles in metabolic homeostasis, specific BAs are implicated in the pathogenesis and progression of HCC through diverse molecular mechanisms such as promoting malignant cell proliferation, affecting cell apoptosis, and inducing immune evasion (25–27). Importantly, recent advances in metabolomic research have established BAs profiling as a promising diagnostic tool for the early detection and predicting the risk and of HCC. The application of machine learning algorithms to comprehensive BAs profiling has demonstrated significant diagnostic utility in distinguishing between benign and malignant hepatobiliary strictures with high accuracy (28). In addition, several investigations have demonstrated significant elevations in the circulating concentrations of specific BAs species such as glycocholic acid, taurodeoxycholic acid and taurocholic acid in the serum/plasma of HCC patients compared to healthy control cohorts (29–31). A study conducted by Wang et al. found that elevated total serum BAs levels were an independent risk factor for HCC development (32). The above studies underscore the pivotal role of BAs profiling in both HCC screening and prognostic prediction, highlighting its potential as a valuable biomarker in clinical hepatology.

It has been recognized that neoplastic tissues constitute complex ecosystems composed of malignant cells and their associated TME. The dynamic interplay between diverse immune cell populations within the TME plays a pivotal role in orchestrating tumorigenesis and modulating cancer (12). The recent advancements in immunotherapeutic strategies have revolutionized the therapeutic landscape for HCC, enhancing treatment efficacy and patient outcomes (33). Given that BAM in the TME is dynamic, the prognostic model constructed based on BAM-related genes involved in the dynamic process to discriminate the risk level of HCC patients holds the promise for facilitating the formulation of personalized treatment strategies and enhancing prognostic assessment in clinical practice. As such, in the present study, we try to explore the influence of BAM-related genes on both the immune-infiltrated TME and their prognostic implications in patients with HCC.

In this study, we identified 111 BAM-related DEGs of HCC from the MSigDB database. We then conducted consensus clustering analysis utilizing BAM-related gene signatures, which enabled the identification of two distinct molecular subtypes. The subtypes exhibited significantly divergent clinical outcomes and immune features in HCC. In this section, we designated the TCGA dataset as the training cohort and the GSE14520 dataset as the validation cohort. While the consensus clustering approach may lack sufficient precision to directly inform immunotherapy strategies, the molecular subtypes derived from BAM-related genes expression profiles demonstrate significant potential for guiding clinical decision-making in HCC management. Survival analysis revealed a statistically significant reduction in overall survival among patients exhibiting low expression levels of BAM-related genes compared to those with high expression profiles.

Through univariate regression analysis, we identified 13 survival-related genes associated with BAM. Subsequently, we constructed a prognostic model using LASSO regression, which identified a four-gene signature comprising AKR1D1, CYP7A1, FABP6, and NPC1. Meanwhile, the patients were divided into high and low-risk groups. We also systematically investigated the differential characteristics between two groups in terms of overall survival, tumor immune infiltration and somatic mutation status. Our findings demonstrate that the risk stratification score exhibits significant correlations with OS and multiple clinical parameters including gender, tumor stage, and T classification. The risk score can serve as an independent prognostic indicator for HCC patients. Moreover, the risk score is associated with immune cells based on CIBERSORT, TIMER, QUANTISEQ, MCP-COUNTER, EPIC, xCell, and CIBERSORT-ABS algorithms. Specifically, the CIBERSORT algorithm analysis demonstrated a positive correlation between the risk score and infiltration levels of Macrophages M0 and Monocytes. Furthermore, GSEA analysis showed that pathways enriched in the high-risk group were mainly related to amino acid catabolic process, fatty acid metabolism and drug metabolism, etc. Pathways enriched in the low-risk group were primarily involved in cytokine-cytokine receptor interaction, ECM-receptor interaction and Hedgehog signaling pathway. In addition, we assessed the differences in ICD- and ICP-related genes between the risk groups and identified statistically significant variations in multiple genes and found that ICP-based immunotherapy may yield differential responses in patients from distinct risk groups.

The Niemann-Pick C1 (NPC1) protein is a 1278-amino acid transmembrane glycoprotein that predominantly localizes to the limiting membrane of late endosomes and lysosomes (34). The NPC1 plays a crucial role in maintaining cellular cholesterol homeostasis by promoting the export of cholesterol from endolysosomes (35). Recently, emerging evidence has revealed elevated NPC1 expression across various malignancies such as breast cancer, gastric cancer, liver cancer etc (36–38). We analyzed the expression of four BAM-related genes across the TCGA, GSE14520, and GSE76427 cohorts and found that only NPC1 was the high expressed gene and demonstrated statistically difference between HCC and normal liver tissues. In light of these discoveries, we performed a series of *in vitro* experimental validations to substantiate the aforementioned findings. Our functional studies demonstrated that NPC1 knockdown suppressed cell proliferation, migration, and invasive capabilities in HepG2 cells.

Our investigation evaluated the model’s predictive efficacy across survival outcomes, clinical and molecular mechanisms, tumor mutational burden as well as the immune landscape. Nonetheless, limitations within our research remain. Firstly, the majority of our analyses were predicated on publicly accessible databases, underscoring the imperative for enhanced clinical validation to substantiate our findings. Secondly, the predominance of bioinformatics-based analyses in our study necessitates further corroboration through fundamental experimental approaches. Furthermore, additional *in vivo* studies and clinical investigations are warranted to elucidate the therapeutic potential of NPC1 and delineate its precise molecular pathways in HCC pathogenesis.

To summarize, through comprehensive bioinformatics analysis, this study established a robust risk model incorporating four BAM-associated genes, which demonstrates high predictive accuracy for HCC patient survival and effectively characterizes the immunological and mutational landscape of HCC. Moreover, we found that silencing NPC1 gene could inhibit HCC cells proliferation, migration, and invasion *in vitro* study. As such, NPC1 can be regarded as a valuable tool for risk stratification and personalized treatment strategies in HCC patients. Collectively, these findings offer valuable perspectives for advancing immunotherapeutic strategies in HCC management.

## Data Availability

The original contributions presented in the study are included in the article/**Supplementary Material**. Further inquiries can be directed to the corresponding authors.
