# Supplementary material for: The role of bile acid metabolism-related genes in prognosis assessment of hepatocellular carcinoma and identification of NPC1 as a biomarker
Source: Front Endocrinol (Lausanne). 2025 May 29;16:1588529. doi: 10.3389/fendo.2025.1588529 (PMC12158721; doi:10.3389/fendo.2025.1588529)
Supplement: Supplementary file 2 [file Table2.docx]

**Table S2. The primers of NPC1 for qPCR**

| **Gene** | **Sequence(5’→3’)** |
| --- | --- |
| NPC1-F | GCACCTTTTACCATCACTCCTG |
| NPC1-R | GGCCACAGACAATAGAGCAGT |
